# Lights and Shadows of Essential Oil-Derived Compounds: Antimicrobial and Anti-Inflammatory Properties of Eugenol, Thymol, Cinnamaldehyde, and Carvacrol

**DOI:** 10.3390/cimb47110915

**Published:** 2025-11-04

**Authors:** Rocco Latorre, Maria Chiara Valerii, Marco Benati, Russell Edward Lewis, Renato Spigarelli, Alberto Bernacchi, Giuseppe Lippi, Enzo Spisni, Paolo Gaibani

**Affiliations:** 1 NYU Pain Research Center, New York University, New York, NY 10012, USA; usa.rl3423@nyu.edu; 2 Department of Molecular Pathobiology, New York University College of Dentistry, New York, NY 10010, USA; 3 Department of Biological, Geological and Environmental Sciences, University of Bologna, Via Selmi 3, 40126 Bologna, Italy; mariachiara.valerii2@unibo.it (M.C.V.);; 4 Section of Clinical Biochemistry, Department of Engineering for Innovative Medicine (DIMI), University of Verona, Piazzale L.A. Scuro 10, 37134 Verona, Italy; marco.benati@univr.it (M.B.);; 5 Department of Molecular Medicine, University of Padova, 35122 Padova, Italy; 6 Microbiology and Virology Unit, Azienda Ospedaliera Universitaria Integrata Di Verona, 37134 Verona, Italy; 7 Microbiology Section, Department of Diagnostics and Public Health, Verona University, 37134 Verona, Italy

**Keywords:** antimicrobials, anti-inflammatory, multidrug-resistance (MDR), Gram-positive, Gram-negative

## Abstract

Essential oil-derived compounds such as eugenol, thymol, cinnamaldehyde, and carvacrol exhibit potent antimicrobial and anti-inflammatory properties, making them promising candidates for therapeutic and industrial applications. This review examines the current evidence regarding the mechanisms of action, efficacy, and ability to disrupt quorum sensing and biofilm formation of essential oil-derived compounds against a broad spectrum of Gram-positive and Gram-negative bacteria, including multidrug-resistant (MDR) strains. The anti-inflammatory activity of these compounds is also highlighted, with emphasis on their modulation of key signaling pathways such as nuclear factor-kappa B (NF-κB) and mitogen-activated protein kinases (MAPKs), and their ability to downregulate pro-inflammatory cytokines. However, challenges persist, including cytotoxicity at high concentrations, chemical instability, poor water solubility, and variable pharmacokinetics. Advanced delivery systems such as nano encapsulation and synergistic formulations offer potential strategies to overcome these limitations. This review highlights both the therapeutic potential and the current limitations of these natural compounds, emphasizing the need for continued research to translate preclinical findings into clinical applications.

## 1. Introduction

**Figure 1:**
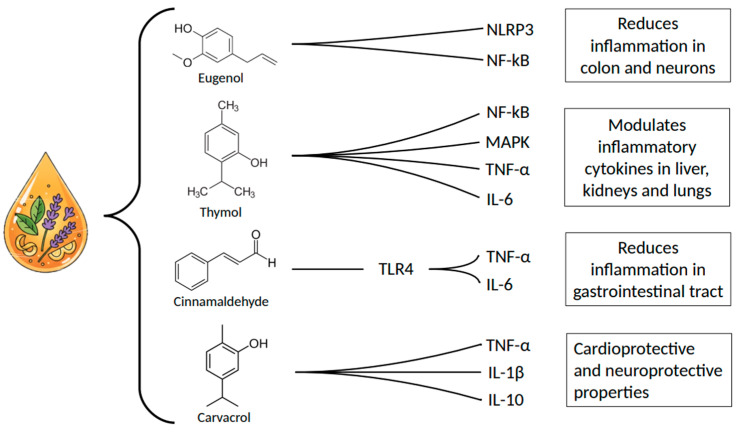
The wide range of biological activities of essential oils.

Essential oils are volatile aromatic compounds extracted from plants that have been widely used in both traditional medicine and modern therapeutic applications because of their powerful bioactive properties. These natural products contain essential molecules including phenylpropanoids such as eugenol and cinnamaldehyde, and phenolic monoterpenes such as thymol, and carvacrol. These molecules in particular have shown significant antibacterial, anti-inflammatory, and antioxidant effects in numerous scientific studies [1,2,3] (Figure 1). However, their integration into evidence-based medicine remains complex. In this review, we refer to this dual nature as “Lights and Shadows”: the *lights* represent their therapeutic potential, including broad-spectrum antimicrobial activity, modulation of inflammatory pathways, and synergy with conventional drugs; the *shadows* reflect the scientific and clinical challenges that limit their wider application. These include variability in chemical composition between batches, instability of volatile components, poor aqueous solubility and bioavailability, potential toxicity at higher doses, and a lack of large-scale, well-controlled clinical trials. By using this framework, we aim to critically examine both the strengths and limitations of essential oils, offering a balanced perspective on their current and future roles in antibacterial and anti-inflammatory therapy.

Eugenol, thymol, carvacrol and cinnamaldehyde exhibit potent antibacterial properties through multiple mechanisms, making them effective against a broad range of pathogenic bacteria. These compounds primarily target bacterial cell membranes, increasing their permeability and leading to the loss of essential intracellular components, ultimately causing cell death [2]. Additionally, inhibiting key bacterial enzymes such as ATPases, essential oils disrupt bacteria energy metabolism and impair survival [1]. Their ability to interfere with quorum sensing and biofilm formation enhances antimicrobial efficacy, especially against resistant bacterial strains like *Pseudomonas aeruginosa* and *Staphylococcus aureus* [4]. Eugenol has demonstrated strong activity against *Escherichia coli*, *Staphylococcus aureus*, and *Listeria monocytogenes*, making it particularly valuable in food preservation and dental applications [5]. Thymol has shown efficacy against *Salmonella*, *Bacillus cereus,* and *Helicobacter pylori*, targeting their enzymatic pathways and preventing bacterial proliferation [6]. Carvacrol, recognized for its broad-spectrum activity, effectively inhibits the growth of *Clostridium difficile*, *Enterococcus faecalis*, and *Acinetobacter baumannii* by disrupting membrane integrity and ion homeostasis [7]. These diverse antibacterial properties underscore the potential of eugenol, thymol, and carvacrol as natural antimicrobial agents in medical and industrial settings. Furthermore, eugenol, thymol, and carvacrol exhibit significant anti-inflammatory properties by modulating key inflammatory pathways and reducing oxidative stress. Eugenol effectively inhibits the enzymes cyclooxygenase (COX) and lipoxygenase (LOX), leading to decreased production of eicosanoids such as prostaglandins and leukotrienes, which are critical mediators of inflammation [8]. For pathological conditions such as colitis and arthritis, eugenol has shown therapeutic potential by reducing inflammatory cytokine levels and minimizing tissue damage [8]. Thymol exerts anti-inflammatory effects by modulating histamine release and inhibiting the activation of nuclear factor-kappa B (NF-κB), which is a well-known central regulator of pro-inflammatory gene expression, leading to decreased levels of TNF-α, IL-6, and IL-1β [9]. Studies have demonstrated that thymol attenuates lung inflammation in asthma models by reducing eosinophilic infiltration and oxidative damage [8]. Carvacrol further strengthens the anti-inflammatory potential of these essential oil constituents by suppressing the NF-κB pathway and functioning as a potent free radical scavenger, mitigating oxidative stress and inflammatory damage [10]. Its neuroprotective effects have been observed in models of neuroinflammation, such as in Parkinson’s disease, where it alleviates inflammation-induced neuronal damage [10]. In addition to eugenol, thymol, and carvacrol, cinnamaldehyde is another key essential-oil constituent with well-documented antimicrobial and anti-inflammatory properties [11,12,13]. For instance, cinnamaldehyde reduces bacterial membrane potential and increases permeability, leading to cell lysis, and impairs quorum sensing and biofilm stability [13]. Derivatives of cinnamaldehyde have been developed that enhance antibacterial potency and selectivity, including those targeting the bacterial cell-division protein FtsZ in multidrug-resistant pathogens [14]. On the anti-inflammatory side, cinnamaldehyde modulates NF-κB signaling and oxidative stress pathways, which may contribute to its therapeutic effects in inflammatory disorders [15]. Given these properties, adding cinnamaldehyde to the scope of our review strengthens the comprehensive perspective on natural phenylpropenes with dual antimicrobial and anti-inflammatory roles. Collectively, essential oil compounds offer promising natural alternatives for managing inflammatory conditions by targeting multiple molecular pathways involved in the inflammatory response. Recent advancements in formulation science have enabled the incorporation of eugenol, thymol, and carvacrol into pharmaceutical and cosmetic products. This integration is supported by extensive research and analytical techniques, such as gas chromatography–mass spectrometry (GC-MS), which allow for precise identification and application of these compounds. Their natural origin makes them an attractive subject of research within the context of developing sustainable therapeutic agents with potentially lower environmental impact. Essentially, these compounds often work together, enhancing their individual effects to achieve greater efficacy. This synergy not only boosts their antibacterial and anti-inflammatory properties but also reduces the side effects that may occur with the use of synthetic drugs. Essential oil compounds like eugenol, thymol, and carvacrol have significant therapeutic potential; however, they also face several limitations that need to be addressed for wider clinical use. High concentrations of eugenol can cause cytotoxicity and hepatotoxicity, which limits its therapeutic window [5]. Although thymol and carvacrol are effective, they may cause mucosal irritation and allergic reactions, especially with prolonged exposure [16]. Stability issues further complicate their application, as these volatile compounds can degrade when exposed to heat and light, thus lowering their effectiveness [17]. Their low solubility in water also limits their bioavailability, necessitating the development of innovative delivery systems, such as nanoencapsulation and liposomal formulations, to enhance absorption and improve therapeutic potential. Additionally, while these molecules disrupt bacterial membranes, extended use may lead to bacterial adaptation through mechanisms like efflux pumps, which could diminish their long-term effectiveness [18]. Addressing these challenges requires advanced formulation strategies, synergistic combinations with conventional antibiotics or anti-inflammatory agents, and rigorous clinical trials to establish safe and effective dosing. Future research should also focus on elucidating their precise molecular mechanisms to optimize the pharmacological applications. Their integration into medicine and healthcare underscores the enduring connection between scientific innovation and natural resources, paving the way for sustainable and effective treatments in the future. In this review, we summarize the antibacterial and inflammatory activities of eugenol, thymol, cinnamaldehyde and carvacrol, and discuss the critical aspects regarding the potential use of these molecules in clinical practice.

## 2. Materials and Methods

### Search Strategy

We searched PubMed, Scopus, and Web of Science for original research articles published over the last 25 years to compile a comprehensive sequence of events regarding the scientific and medical use of essential oils. Our search utilized terms such as “eugenol,” “thymol,” “cinnamaldehyde,” and “carvacrol,” combined with keywords like “antimicrobial,” “anti-inflammatory,” “NF-κB,” “Nrf2,” “ROS,” “TRP,” “membrane,” “biofilm formation,” “synergistic effects,” and “toxicity.” We included in vitro, in vivo, and formulation studies published in English. Additionally, we consulted and cited reviews and meta-analyses for their significance in summarizing key points, while also referencing primary sources to ensure we had the most up-to-date information.

## 3. Narrative Description

### 3.1. Eugenol

Eugenol (4-allyl-2-methoxyphenol) consists of a benzene ring bearing a hydroxyl, a methoxyl, and an allyl group, the latter contributing to its lipophilic properties [18]. Investigations into its antibacterial mechanisms have revealed that its primary mode of action involves the disruption of bacterial cell membranes, leading to the leakage of intracellular contents and subsequent cell death [10,19]. However, some authors suggest that its effectiveness can be influenced by environmental factors such as temperature, pH, and the presence of other compounds [17,18,19,20], as well as by the variability among bacterial strains [19,20,21,22]. Generally, eugenol yields minimum inhibitory concentration (MIC) values ranging from 0.5 to 1 mg/mL against Gram-negative bacteria such as *Escherichia coli*, *Pseudomonas aeruginosa*, and *Klebsiella pneumoniae* [5], while values ranging from 0.8 to 1.75 mg/mL have been reported for Gram-positive bacteria (Table 1) [5].

The effects of eugenol are mainly related to its ability to damage cellular membranes, thereby altering their permeability. For this reason, eugenol can restore drug sensitivity in many multidrug-resistant species, including *Pseudomonas aeruginosa* [27] and multidrug-resistant (MDR) *Klebsiella pneumoniae* strains, which are associated with severe infections and high mortality rates [28]. Eugenol also exhibits remarkable anti-biofilm properties: when tested on MDR *Staphylococcus aureus*, it was able to inhibit biofilm formation and disrupt pre-existing biofilms by damaging cell-to-cell connections [24]. Moreover, eugenol can interfere with quorum-sensing, reducing the production of virulence factors and biofilm-promoting elements in toxigenic bacteria [29] as well as in fungal species such as *Candida albicans* [30].

The anti-inflammatory properties of eugenol have also been investigated in a model of lipopolysaccharides (LPS)-induced colon inflammation in mice. In this study, eugenol was able to reduce colonic inflammation by modulating the NLRP3 (NLR family pyrin domain containing 3) inflammasome pathway [31] and inhibiting the nuclear NF-κB pathway [32]. Moreover, based on other studies, its anti-inflammatory effects may extend to neuroinflammatory conditions associated with neurodegeneration [33] and hepatoprotection [34]. However, some authors have highlighted the potential toxicity of this compound, so that its use as a therapeutic agent should be preceded by a careful definition of dosage and formulation to prevent side effects [35]. Like other essential oil components, discussed further in this review, eugenol is a potent modulator of reactive oxygen species (ROS) and transient receptor potential (TRP) ion channels. Eugenol can exert both antioxidant and pro-oxidant effects, depending on the cellular context and concentration. At lower concentrations, eugenol can scavenge free radicals and reduce oxidative stress. However, at higher concentrations, eugenol can induce ROS production, which may contribute to its antimicrobial and cytotoxic effects. Furthermore, eugenol is known to interact with TRP ion channels, a family of sensory receptors involved in pain, temperature, and taste perception. For example, eugenol, with carvacrol and thymol, are known activators of TRPV3, a heat-sensitive channel, which may explain the warm sensation they produce. The interaction of eugenol and other essential oil compounds with TRP channels represents an important and emerging area of research that may explain some of its complex pharmacological effects [31,32,33,34,35].

Pharmacokinetics studies have shown that eugenol effectively crosses biological barriers and distributes into various tissues, including the brain [36]. However, it is also rapidly metabolized into glucuronide and sulfate conjugates [37] and is mainly excreted in the urine [38]. When administered orally, eugenol shows rapid clearance [9,36,38,39,40,41], whereas its formulation into nanocarriers has been shown to enhance its stability [40] and improve its overall availability.

### 3.2. Thymol

Thymol (2-isopropyl-5-methylphenol) is a monoterpenoid phenol with broad-spectrum antimicrobial and anti-inflammatory activities [42,43]. Its hydroxylated benzene ring confers both lipophilic and electron-delocalizing properties, enabling the compound to interact with and destabilize microbial membranes, thereby increasing permeability and leading to cell death through autolysis, cellular stress, and growth inhibition [43,44,45,46,47]. In addition, thymol can interact directly with intracellular proteins and enzymes, disrupting their normal function. For example, thymol has been shown to inhibit NADPH production at a concentration of 200 µg/mL, by targeting an aldo-keto reductase in *Staphylococcus aureus* [46]. Overall, thymol induces differential regulation in expression of proteins involved in thermal and envelope stress, promotes accumulation of misfolded proteins in the outer membrane, and impairs the citrate metabolic pathway [48]. As a result, thymol exhibits strong antimicrobial activity against Gram-positive bacteria such as *Staphylococcus aureus* and *Listeria monocytogenes*, Gram-negative species like *Escherichia coli*, and various pathogenic fungi (Table 2) [43,44,45].

Several studies have shown that thymol can reduce bacterial biofilm formation during both the initial and mature phases [54,55,56] by disrupting the extracellular polymeric substance (EPS) matrix and interfering with bacterial adhesion and communication [54,55]. Thymol effectively eradicates the EPS matrix produced by MDR strains of *Staphylococcus aureus* and *Pseudomonas aeruginosa* [54] and eliminates mature biofilms by decreasing the production of polysaccharide intercellular adhesin and extracellular DNA, which are pivotal for biofilm integrity [56].

Moreover, when combined with other natural compounds commonly found in essential oils, such as carvacrol and eugenol, its antimicrobial efficacy is enhanced even at lower dosages, reducing the risk of microbial resistance [43] and further inhibiting biofilm formation [45]. Its efficacy, stability, and delivery can be improved through nano- and micro-encapsulation [43]. For instance, encapsulation in gelatin methacryloyl-based nanonosomes has been shown to enhance its antibiofilm activity and promote wound healing [57]. Beyond its antimicrobial properties, thymol exhibits significant anti-inflammatory effects both in vitro and in vivo [58,59]. By modulating the NF-κB and mitogen-activated protein kinase (MAPK) pathways, it can reduce the expression of pro-inflammatory cytokines and the phosphorylation of p38 and Jun N-terminal kinases (JNK) [58]. Furthermore, thymol decreases the production of inflammatory mediators such as interleukin (IL)-6 and tumor necrosis factor (TNF)-α [43,59], interfering not only with the initiation of the inflammatory response but also with its propagation. Thymol has been shown to ameliorate ischemic brain injury by inhibiting microglial activation and reducing inflammatory interleukins [39], as well as to reduce apoptosis and lysosomal stress in LPS-induced acute kidney and liver inflammation [60,61]. An interesting overview of the Thymol effect on inflammation was given by recent studies, which have elucidated a novel mechanism by which thymol exerts its anti-inflammatory effects in the lungs. Specifically, thymol has been shown to activate the nuclear factor erythroid 2–related factor 2 (Nrf2)/heme oxygenase-1 (HO-1) signaling pathway. Nrf2 is a transcription factor that plays a critical role in the cellular antioxidant response. Its activation by thymol leads to the upregulation of HO-1, an enzyme with potent anti-inflammatory and antioxidant properties. This Nrf2-mediated antioxidant response is crucial for protecting lung tissue from oxidative damage during inflammation. Furthermore, thymol has been found to inhibit the activation of nuclear factor-kappa B (NF-κB) induced by lipopolysaccharide (LPS) in lung injury models. NF-κB is a key regulator of the inflammatory response, and its inhibition by thymol contributes to the downregulation of pro-inflammatory cytokines such as TNF-α and IL-6 [34,60].

Thymol is one of the few essential oil compounds that is solid at room temperature. Therefore, solvents are usually used for its administration. As for its pharmacokinetic properties, the absorption of thymol is strongly influenced by its lipophilicity, which facilitates membrane permeability but also drives preferential accumulation in lipid-rich tissues such as the plasma, liver, kidneys, and large intestine. Consequently, its distribution is uneven, with low concentrations detected in muscle tissue [62]. Oral administration results in variable absorption rates, also depending on solvents used, and significant first-pass metabolism [63]. Thymol is primarily metabolized through phase II conjugation, leading to the formation of thymol sulfate and thymol glucuronide, which are more hydrophilic and readily excreted [64]. These pathways contribute to its short plasma half-life and generally low systemic concentrations, which may limit its therapeutic efficacy for systemic infections or inflammation [40,64]. To overcome these apparent limitations, several formulation and delivery strategies have been proposed. Encapsulation of thymol in liposomes, solid lipid nanoparticles (SLNs), or nanostructured lipid carriers (NLCs) can protect it from premature metabolism, enhance aqueous dispersibility, and provide controlled release, leading to improved bioavailability and prolonged plasma retention [65]. Formation of thymol–cyclodextrin complexes can increase water solubility and stability, improve oral absorption, and reduce volatility [62]. Targeted delivery to the intestine can bypass part of the first-pass metabolism and increase local concentrations in gastrointestinal disorders while minimizing systemic toxicity, using mucoadhesive and enteric-coated formulations [65]. Co-administration with metabolic enzymes, such as safe phase II metabolism modulators (e.g., quercetin), could enhance systemic exposure and finally transdermal and intranasal delivery systems, to bypass hepatic first-pass metabolism and provide sustained therapeutic plasma levels for systemic effect [64].

By integrating such advanced delivery approaches, it is possible to improve thymol’s pharmacokinetic profile, reduce dose variability, and expand its therapeutic utility beyond local antimicrobial and anti-inflammatory effects. These strategies could pave the way for clinically relevant, standardized thymol-based interventions.

### 3.3. Cinnamaldehyde

Cinnamaldehyde is a naturally occurring aromatic aldehyde with molecular formula C_9_H_8_O. It consists of a benzene ring bearing an α,β-unsaturated aldehyde group. Trans-cinnamaldehyde predominantly adopts the s-trans conformation and is generally more abundant than the cis form due to its greater chemical stability [66]. The hydrophobic structure of cinnamaldehyde enables its interaction with microbial cell membranes, resulting in easy penetration of the cell wall of bacteria and fungi, intercalation into the lipid bilayer, and an increase in membrane fluidity and permeability. This disruption of the cell membrane leads to the leakage of essential intracellular components such as ions, ATP, and nucleic acids [67,68] and inhibition of key enzymatic pathways [69], ultimately resulting in cell death. This mode of action is also seen in other essential oils such as thymol and eugenol. Cinnamaldehyde is proven effective against both Gram-negative and Gram-positive bacteria (Table 3). Reported MIC values range from 100 to 400 µg/mL for Gram-negative species, and from 800 to 1750 µg/mL for Gram-positive bacteria [26]. This difference suggests that its antimicrobial efficacy is strongly influenced by the structural characteristics of the bacterial cell envelope [26].

The antimicrobial potential of cinnamaldehyde has also been explored in veterinary sciences, where it has shown efficacy against mastitis-associated *Aggregatibacter* strains [71] and demonstrates promising effects against MDR pathogens, especially when used in combination with other antimicrobial agents. For instance, cinnamaldehyde appears to reduce the expression of extended-spectrum β-lactamases (ESBLs) in MDR *Salmonella* strains, thereby enhancing the antibacterial activity of ceftriaxone [72]. Several studies have confirmed the ability of this compound to inhibit resistance mechanisms in MDR bacteria, thus restoring their susceptibility to conventional antibiotics. Mireles et al. (2024) [73] described how cinnamaldehyde can reverse colistin resistance in *Enterobacterales* and *Acinetobacter baumannii*. These effects appear to be enhanced when cinnamaldehyde is used synergistically with other natural compounds. Cinnamon bark oil, whose main component is cinnamaldehyde, exhibits stronger antibacterial activity against *Pseudomonas aeruginosa* than cinnamaldehyde alone [74].

Cinnamaldehyde properties extend beyond direct microbial inhibition, as it can disrupt biofilm architecture and quorum sensing mechanisms, which are critical for the virulence of many pathogenic microorganisms [75,76]. Cinnamaldehyde and its structural analogues have been actively tested against uropathogenic *Escherichia coli* and *Staphylococcus aureus* [77], highlighting the importance of its chemical structure, particularly the α,β-unsaturated aldehyde group, which plays a central role in inhibiting biofilm formation.

In methicillin-resistant *S. aureus* (MRSA), cinnamaldehyde was able to reduce biofilm viability and biomass at concentrations five times lower than the MIC [78]. In *Pseudomonas aeruginosa*, cinnamaldehyde disrupted quorum-sensing mechanisms involved in biofilm formation [79] and reduced the expression of virulence factors. Similar effects were observed in *Streptococcus* spp., where cinnamaldehyde increased surface hydrophobicity, reduced cellular virulence, decreased biofilm mass accumulation, and altered quorum-sensing signals [80].

In addition to its antimicrobial effects, cinnamaldehyde can modulate inflammatory pathways, for instance by inhibiting Toll-like receptor 4 (TLR4), thereby reducing the expression of IL-6 and TNF-α [6,81]. Both in vitro [81,82] and in vivo [83,84] studies have shown that this compound is also capable of reducing the production of ROS, thus attenuating the activation of the NF-κB pathway. The moderate lipophilicity and low molecular weight of cinnamaldehyde are likely to facilitate its absorption across biological membranes following oral administration [85], allowing for efficient gastrointestinal uptake [86].

In terms of metabolism, cinnamaldehyde undergoes phase I oxidation (yielding cinnamic acid derivatives) and phase II conjugation, which support both biliary and renal elimination [85,86]. However, quantitative data on its excretion remain poorly established. Given its potent antimicrobial, anti-inflammatory, and anti-biofilm activities, coupled with a favorable safety profile at appropriate doses, cinnamaldehyde holds considerable promise as a preventive agent against infections and inflammation, warranting further well-designed clinical studies to translate these preclinical benefits into human health applications [87].

### 3.4. Carvacrol

Carvacrol (2-methyl-5-(1-methylethyl) phenol) is a natural occurring mono-terpenoid phenol that is commonly found in oregano and thyme essential oils [88]. Its lipophilic molecular structure, characterized by a free hydroxyl group attached to an aromatic ring, facilitates the interaction with microbial cell membranes, disrupting their integrity [89], leading to uncontrolled leakage of vital intracellular components [89,90]. Thus, carvacrol is effective against fungi, including *Aspergillus* spp. and *Candida* spp. [90] (Table 4).

These antimicrobial properties are extended to multidrug-resistant strains of pathogenic bacteria, since Carvacrol can inhibit the growth of nosocomial methicillin-resistant strains of *Staphylococcus aureus* (MRSA) at concentrations ranging between 0.03 and 0.015% (*v*/*v*) when diluted in DMSO [92]. In vivo studies on MDR *Salmonella enterica* inoculated celery and oysters, showed that 1% carvacrol sensitively reduced the bacterial population [93], underlining the effects of this compound both in vivo and in vitro. Moreover, carvacrol is effective in reducing the resistance of MDR *Salmonella thyphimurium*, *Streptococcus pyogenes* and *S. aureus* strains [94]. Carvacrol can also disrupt the bacteria’s communication system with its anti-quorum sensing and anti-biofilm formation characteristics [95,96] resulting in synergistic activity with other antimicrobial compounds (i.e., cefixime and thymol) [81,97,98]. For instance, sub-lethal concentrations of this compound inhibited quorum sensing mechanisms in *Chromobacterium violaceum*, leading to a significant reduction in biofilm formation [1] and daily exposure to a 20 mM carvacrol solution could inhibit *S. aureus* biofilm accumulation in fermentor systems [1]. Although the antimicrobial effect against *Staphylococcus aureus* has also been reported by Knowles et al. [99], the compound appears to lose its efficacy when applied at the same concentration and exposure time to mature *Salmonella* spp. biofilms. Notably, these biofilms, which were susceptible to treatment in their early developmental stages, exhibit increased resistance at maturity [100,101]. This reduced efficacy is likely due to the mechanism of action of carvacrol, which includes the inhibition of flagella synthesis, thereby preventing initial bacterial surface attachment [102]. However, once a biofilm is fully established, carvacrol appears unable to penetrate the biofilm matrix effectively or disrupt the bacterial cell membranes within [102]. Since the discovery of techniques that improve the stability, delivery and efficacy of carvacrol (i.e., nano-encapsulation) [98,103,104], this molecule has been used in active food packaging to inhibit food-borne pathogens such as *Escherichia coli*, *Salmonella*, and *Bacillus cereus* [105] and for biomedical applications [104]. When encapsulated in soluble potato starch nanofibers at a concentration of 30% (*v*/*v*), carvacrol can inhibit *Listeria monocytogenes*, *S. typhimurium* and *E. coli* [106]. Beyond its antimicrobial actions, carvacrol has also shown anti-inflammatory properties [107,108], being able to inhibit pro-inflammatory mediators as IL-1β and TNF-α [109], while enhancing anti-inflammatory cytokines like IL-10 [108,109]. Moreover, it has been reported to limit tissue damage by reducing the release of neutrophil elastase [110]. These anti-inflammatory effects are significant for medical applications such as wound healing [111], treatment of respiratory disorders [112], and protection against myocardial injury and neuroinflammation [113,114]. Pharmacokinetic studies indicate that carvacrol has a relatively long half-life and low clearance following intravenous administration [115]. These properties are further enhanced when the compound is incorporated into nanocarrier systems [115]. Its bioactivity and therapeutic duration can be significantly affected by phase I metabolic reactions, such as oxidation of its methyl and isopropyl groups [116], as well as degradation processes occurring in the gastrointestinal tract [117].

### 3.5. Therapeutic Potential and Clinical Considerations

The clinical development of essential oils as systemic antimicrobials typically faces several obstacles. Despite the growing interest in their therapeutic potential, the medical application of essential oils remains controversial due to multiple scientific, clinical, and regulatory challenges. One of the most significant issues is the lack of standardization in the chemical composition, which can vary depending on plant species, cultivation, harvest time, extraction method, and storage conditions [118]. This variability complicates reproducibility and undermines efforts to establish consistent dosing regimens. Safety concerns persist as well. Certain constituents, particularly eugenol, thymol, and cinnamaldehyde, have demonstrated cytotoxicity, hepatotoxicity, and genotoxicity at higher concentrations or with prolonged exposure [35]. Such effects are often underreported in commercial or alternative medicine contexts, potentially leading to misuse or overuse. Furthermore, interpretation of antimicrobial potency data is complicated by variability in experimental conditions and formulations. The use of different solvents, emulsifiers, or nano-carrier systems can significantly alter compound stability, solubility, and bioavailability, leading to inconsistent MIC values across studies. These formulation-dependent differences make it difficult to directly compare potency data between laboratories or species models and underscore the need for standardized testing conditions and formulation reporting in future research. Based on data from the Joint FAO/WHO Expert Committee on Food Additives (JECFA) and the European Food Safety Authority (EFSA) obtained from acute, subchronic, and chronic toxicity studies in rats, eugenol shows an acute toxicity (Lethal Dose, 50%, LD_50_) of approximately 1930 mg/kg, while chronic/subchronic studies indicate a NOAEL (No Observed Adverse Effect Level) of about 300 mg/kg day. The ADI (Acceptable Daily Intake) is 0–2.5 mg/kg bw day [119,120,121]. For cinnamaldehyde, the oral LD_50_ is reported to be ~2220 mg/kg, with a NOAEL of ~125–250 mg/kg day [119,121]. For thymol, LD_50_ is ~980 mg/kg and NOAEL ~200–250 mg/kg day [119,122,123]. For carvacrol, the oral LD_50_ is ~810 mg/kg and the NOAEL ~200 mg/kg day [119,122,123]. For these last three compounds, a formal ADI has not been established; however, regulatory evaluations (JECFA) conclude “no safety concern at current flavor use levels.” [124]. In addition, EFSA, in the context of feed additives, indicates safe species-specific levels for thymol in the order of tens to hundreds of mg/kg feed [123]. At doses above those considered safe, the main adverse effects reported are hepatotoxicity (eugenol, carvacrol), nephrotoxicity (thymol, carvacrol), and mucosal/irritative lesions (cinnamaldehyde, thymol). Acute toxicity causes gastrointestinal and neurological disturbances (dizziness, convulsions), as well as hepatic and renal injury. These findings underscore the need to maintain clinical (or experimental) exposures well below the levels that induce toxicity, and to prefer strategies that maximize local effects while reducing systemic absorption. The in vitro studies reported in this review show antibacterial and anti-biofilm activity at medium-to-high concentrations, with MIC values up to 4 mg/mL for eugenol, 475 µg/mL for thymol, ~200 µg/mL for carvacrol against several Gram-positive strains, and as high as 8–10 mg/mL for *S. aureus*. For cinnamaldehyde, MIC values ranged from 12.5 µg/mL (*Enterococcus*, *Salmonella*, *E. coli*) up to 800–1750 µg/mL against resistant Gram-positive strains. These values show high variability and could sometimes be overestimated depending on the method used. Anyway, based on these data, therapeutic plasma levels may not be achievable in vivo within an acceptable safety margin. Moreover, these compounds display poor bioavailability due to limited intestinal absorption and rapid metabolism, mainly glucuronidation and sulfation [36] or oxidation in the case of aldehydes [117] which could further increase the therapeutic dose and thereby the risk of hepatic and renal toxicity. Therefore, clinical translation would require careful dose evaluation and targeted formulations, including the use of delivery systems that maximize local exposure while avoiding systemic peaks, as well as topical or tissue-targeted administration, or synergistic strategies that allow robust dose reduction.

Another limitation is that the antimicrobial activity of many essential oils is elicited through a combination of poorly defined compound interactions at the bacterial cell membrane (at micromolar concentrations). The absence of well-defined molecular targets presents a significant barrier to the rational development of essential oil-derived compounds as therapeutics. Most of their bioactivity arises from multi-component and membrane-level interactions, rather than specific ligand–receptor or enzyme–inhibitor relationships. This mechanistic complexity complicates the identification of pharmacologically relevant targets and limits the applicability of traditional medicinal-chemistry optimization strategies. As a result, structure-activity relationship analyses and potency tuning are challenging, hindering the translation of these molecules into clinically viable drug candidates. Future studies integrating biophysical membrane assays, omics-based profiling, and computational docking approaches could help elucidate target pathways and guide next-generation compound design. Therefore, classical iterative approaches used in medicinal chemistry and clinical pharmacology for optimizing the potency and safety of compounds prior to first-in-human testing are difficult to execute, especially when the specific drug target and inhibitor are not well characterized, as this knowledge is essential for guiding structure-activity relationship optimization and enhancing molecular specificity. For all these reasons, clinical evidence supporting the efficacy of essential oils in human populations remains limited. While in vitro and in vivo animal studies demonstrate promising antimicrobial, anti-inflammatory, and antioxidant properties, well-designed randomized controlled trials (RCTs) in humans are scarce and often small in scale [81,125]. Regulatory agencies such as the Food and Drug Administration (FDA) and the European Medicines Agency (EMA) generally classify essential oils as food additives or cosmetic ingredients rather than as drugs, further complicating their clinical adoption [126]. Other potential drawbacks include the risk of allergic reactions and skin sensitization, particularly when used topically or through inhalation in enclosed spaces [127]. Pharmacokinetic interactions are also a concern, as these compounds could modulate cytochrome P450 enzymes, potentially altering the metabolism of co-administered drugs [128]. Finally, patent limitations on naturally occurring compounds reduce commercial incentives for large-scale clinical study and pharmaceutical development.

Despite these limitations, essential oils retain substantial promise for pharmaceutical use in humans, particularly as adjunctive or topical therapies for infections and inflammation. Their multi-targeted mechanisms, including membrane disruption, quorum sensing inhibition, anti-biofilm activity, and modulation of inflammatory signaling pathways, are advantageous against multidrug-resistant bacteria and chronic inflammatory conditions [125,126,127,128,129]. Clinical data, though still limited, demonstrate efficacy in certain applications: tea tree oil and thyme oil have shown benefits in topical bacterial infections [129]. Essential oil–based mouth rinses reduce dental plaque and gingivitis [130], and oregano or cinnamon oils can attenuate upper respiratory infection symptoms in small controlled trials [131]. Moreover, formulation science is rapidly advancing, with nanoencapsulation, liposomal delivery, and cyclodextrin inclusion complexes improving solubility, stability, and targeted delivery. These approaches may help overcome many pharmacokinetic and stability challenges that have historically hindered systemic use.

In conclusion, while scientific skepticism and regulatory caution are justified, the preclinical and emerging clinical evidence supports continued investigation of essential oils as part of a modern pharmacological toolkit. When rigorously standardized, chemically characterized, and formulated for optimal delivery, essential oils could play a meaningful role in human antibacterial and anti-inflammatory therapy, especially in the context of antibiotic resistance and the need for safer, multi-mechanistic treatment options.

### 3.6. Synergistic Activity of Essential Oils with Antimicrobials Molecules

The in vitro evaluation of the interactions between different molecules has the potential for define therapeutic approaches by suggesting the possible synergistic or antagonistic interaction between different compounds. Indeed, synergy provides valuable information for optimizing antimicrobial therapy, especially for antimicrobials acting on different pathways and between molecules from various sources.

In this context, in recent years, different in vitro studies evaluated the potential synergistic effects of essential oils in combination with different antimicrobial drugs [132,133]. Against Gram-positive bacteria, synergistic interactions of essential oils with different antimicrobials have been reported in limited data in the literature and often reported discordant results [134].

Against Gram-negative microorganisms, thymol and carvacrol exhibited variable synergistic effects in combination with β-lactams by exhibiting synergistic interaction with carbapenem and additive effects in combination with 3rd generation cephalosporins [135,136]. Similarly, eugenol and cinnamaldehyde exhibited synergistic effects in combination with carbapenem (i.e., meropenem), while lower synergistic interaction was observed when tested in combination with other β-lactams [137]. Of note, all these essential oil compounds showed high synergistic interaction when combined with colistin against different Gram-negative pathogens [27,137]. In this context, colistin acts by altering the outer membrane (OM) architecture of Gram-negative microorganisms, thus resulting in an increased permeability of the cell membrane and consequently bacterial death [138]. Consequently, the synergistic interaction between colistin and essential oils has been hypothesized to be associated with a similar bacterial target by inducing an incomplete bacterial membrane, leading to the leakage of the bacterial contents. Although these interactions exhibited promising in vitro results, further in vivo studies are needed to evaluate the clinical applications of these combinations by considering the pharmacokinetics and pharmacodynamics properties of single antibiotics and essential oils.

## 4. Conclusions

Essential oils hold potential as natural antibacterial agents in the battle against pathogens. The different molecular structure of compounds in essential oils accounts for their diverse biological and pharmacological effects. The lipophilic profile of these molecules contributes to their ability to penetrate cells and tissues, reaching biological targets and generating a pharmacological response. The antimicrobial activities of these compounds have been attributed to their ability to penetrate microbial cells, causing structural and functional changes due to their hydrophobic nature. This disruption of the cytoplasmic membrane leads to cell lysis and release of intracellular compounds, resulting in inhibition of key enzymatic pathways. For example, thymol has been shown to inhibit NADPH production by targeting an aldo-keto reductase in *Staphylococcus aureus*, or cinnamaldehyde interacts with the cell membrane and induces rapid inhibition of energy metabolism.

**Figure 2:**
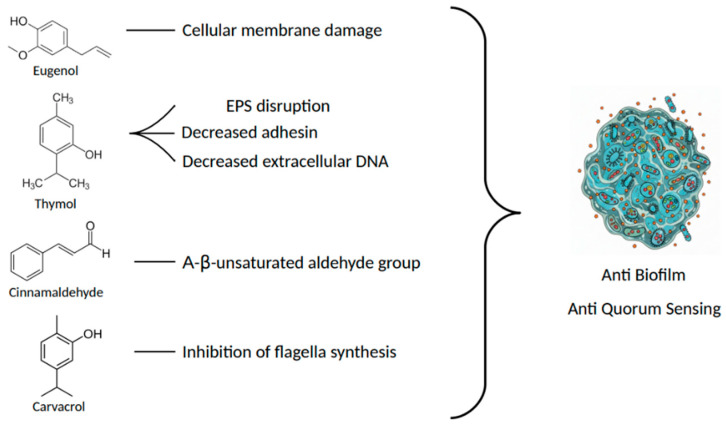
The multiple complementary mechanisms of essential oils in the antimicrobial properties.

The cumulative evidence presented in this review confirms that essential oil-derived compounds, particularly eugenol, thymol, cinnamaldehyde, and carvacrol, possess robust antimicrobial and anti-inflammatory properties supported by a substantial body of in vitro and in vivo evidence. These compounds act through multiple complementary mechanisms, including disruption of microbial membranes, inhibition of quorum sensing and biofilm formation (Figure 2), and modulation of key inflammatory mediators such as NF-κB, IL-6, TNF-α, and IL-1β.

This multi-targeted approach not only enhances their efficacy against a broad range of pathogens, including MDSs, but also underpins their potential use in managing complex inflammatory disorders. Furthermore, we found that almost all essential oils can go beyond direct microbial inhibition, as they can disrupt biofilm architecture and quorum sensing mechanisms that are critical to the virulence of many pathogenic microorganisms. The identification of weak points in the biofilm architecture and the elucidation of important biofilm-specific targets are proving to be crucial focal points for development of targeted therapeutic strategies. Several studies have confirmed the ability of this substance to inhibit resistance mechanisms in MDR bacteria and thus restore their sensitivity to conventional antibiotics. Despite these promising findings, several critical issues limit their clinical translation. First, cytotoxicity remains a significant concern, particularly for eugenol and cinnamaldehyde, which exhibit dose-dependent toxic effects on mammalian cells and organs (e.g., the liver). Second, their physicochemical characteristics, namely volatility, poor aqueous solubility, and chemical instability under light and heat, pose challenges for formulation and long-term storage. These factors, along with rapid metabolism and systemic clearance, significantly constrain their bioavailability and therapeutic window. Furthermore, the variability in reported MIC values across studies reflects not only methodological differences but also genuine strain-dependent efficacy, which complicates the establishment of standardized therapeutic protocols. Lastly, a major limitation of essential oils is the high dosage required to combat resistant microorganisms, which hinders their therapeutic use. The data in this review show that the biological activity depends on the concentration of the essential oil. However, some components of essential oils need to be monitored, and these compounds should be used very carefully due to their toxic effects.

In recent years, nanotechnology-based strategies such as nanoencapsulation, liposomal delivery, and polymeric nanocarriers have shown promise in improving the stability, solubility, and targeted delivery [139,140,141]. Similarly, synergistic combinations with antibiotics or other phytochemicals can enhance antimicrobial efficacy while reducing the risk of resistance and lowering the required effective doses. However, these approaches are largely in preclinical stages and require further investigation through well-designed pharmacokinetic studies and clinical trials to assess safety, efficacy, and optimal dosing in humans. Future research should also prioritize mechanistic studies to dissect the precise molecular interactions of these compounds with microbial and host targets. Particular attention should be paid to their effects on host microbiota and immune modulation, as well as the potential for resistance development with prolonged use. Moreover, standardized models for evaluating biofilm inhibition and anti-inflammatory efficacy, especially in complex disease-relevant settings, are essential for translating laboratory findings into practical medical applications.

While eugenol, thymol, cinnamaldehyde, and carvacrol hold significant therapeutic potential, the realization of this potential will require a multidisciplinary effort combining pharmacology, formulation science, microbiology, and clinical medicine. In this context, there is a need for more well-designed studies involving dose normalization and incubation time in cell and animal models to gain a better understanding of their biological activities and underlying mechanisms. Their future lies not just in their standalone use but as part of integrative therapeutic strategies addressing the growing need for effective, natural, and sustainable antimicrobial and anti-inflammatory solutions.

## Figures and Tables

**Table 1:** MIC values of Eugenol against selected Gram-positive and Gram-negative bacteria.

Gram-Positive	MIC	References
*Listeria monocytogenes*	0.5–1.0 mg/mL	[23]
*Staphylococcus aureus*	1.25–4.0 mg/mL	[19,24]
**Gram-Negative**		
*Escherichia coli*	1–3.0 mg/mL	[25,26]
*Salmonella* spp.	0.2–0.8 mg/mL	[26]

**Table 2:** MIC values of thymol against selected Gram-positive and Gram-negative bacteria.

Gram-Positive	MIC	References
*Staphylococcus aureus*	400 µg/mL–135 mg/L	[49,50]
*Streptococcus mutans*	100 µg/mL	[51]
**Gram-Negative**		
*Salmonella typhimurium*	80 µg/mL	[52]
*Escherichia coli*	45 mg/L	[50]
*Klebsiella pneumoniae*	475 µg/mL	[53]

**Table 3:** MIC values of cinnamaldehyde against selected Gram-positive and Gram-negative bacteria.

Gram-Positive	MIC	Reference
*Bacillus cereus*	3.12 µg/mL	[70]
*Enterococcus faecalis*	0.78 µg/mL	[70]
*Listeria monocytogenes*	6.25 µg/mL	[70]
*Micrococcus luteus*	6.25 µg/mL	[70]
*Staphylococcus aureus*	1.56 µg/mL	[70]
**Gram-Negative**		
*Aeromonas hydrophila*	0.78 µg/mL	[70]
*Escherichia coli*	12.5 µg/mL	[70]
*Escherichia coli* O157:H7	6.25 µg/mL	[70]
*Pseudomonas aeruginosa*	12.5 µg/mL	[70]
*Salmonella enteritis*	6.25 µg/mL	[70]
*Aggregatibacter actinomycetemcomitans*	209 µg/mL	[71]

**Table 4:** MIC values of carvacrol against selected Gram-positive and Gram-negative bacteria as well as both Gram-positive and Gram-negative bacteria, such as *Staphylococcus epidermis*, *Staphylococcus aureus*, *Enterococcus faecalis*, *Klebsiella pneumoniae*, *Escherichia coli*, *Streptococcus pneumoniae*, *Proteus mirabilis*, *Serratia* spp., *Enterobacter* spp. with MIC values ranging from 200 to 50 µg/mL [91]. Activity has also been reported against *Citrobacter diversus*, *Enterobacter amnigenus*, *Proteus mirabilis*, *Providencia rettgeri*, *Pseudomonas aeruginosa* with MIC ranging between 1 and 0.2% (*v*/*v*) [89].

Gram-Positive	MIC	Reference
*Staphylococcus aureus* ATCC 29213	100 µg/mL	[89]
*S. aureus* MRSA WKZ-1	50 µg/mL	[89]
*Enterococcus faecalis* ATCC 29212	100 µg/mL	[89]
*Staphylococcus aureus*	0.2% (*v*/*v*)	[92]
**Gram-Negative**		
*Escherichia coli* ATCC 25922	200 µg/mL	[89]
*Salmonella typhimurium* ATCC 14028	100 µg/mL	[89]
*Acinetobacter baumannii* ATCC 17878	100 µg/mL	[89]
*Citrobacter diversus*	0.2% (*v*/*v*)	[92]
*Enterobacter amnigenus*	0.8% (*v*/*v*)	[92]
*Proteus mirabilis*	0.8% (*v*/*v*)	[92]
*Providencia rettgeri*	0.8% (*v*/*v*)	[92]
*Pseudomonas aeruginosa*	>1% (*v*/*v*)	[92]

## Data Availability

No new data were created or analyzed in this study. Data sharing is not applicable to this article.
